# Predicting Health-Related Quality of Life in Trauma-Exposed Male Veterans in Late Midlife: A 20 Year Longitudinal Study

**DOI:** 10.3390/ijerph17124554

**Published:** 2020-06-24

**Authors:** Samantha M. Stevens, Daniel E. Gustavson, Bin Fang, Xin Tu, Mark Logue, Michael J. Lyons, Chandra A. Reynolds, William S. Kremen, Carol E. Franz

**Affiliations:** 1 Department of Psychiatry, University of California San Diego, La Jolla, CA 92093, USA; sms8402@psu.edu (S.M.S.); daniel.e.gustavson@vumc.org (D.E.G.); bin.fang.0605@gmail.com (B.F.); wkremen@health.ucsd.edu (W.S.K.); 2 Department of Psychology, The Pennsylvania State University, State College, PA 16801, USA; 3 Department of Otolaryngology, Vanderbilt University Medical Center, Nashville, TN 37232, USA; 4 Department of Family Medicine and Public Health, University of California San Diego, La Jolla, CA 92093, USA; x2tu@health.ucsd.edu; 5 Department of Psychological and Brain Sciences, Boston University, Boston, MA 02215, USA; loguem@bu.edu (M.L.); mlyons@bu.edu (M.J.L.); 6 Department of Psychology, University of California Riverside, Riverside, CA 92521, USA; chandrar@ucr.edu; 7 Center of Excellence for Stress and Mental Health, VA San Diego Healthcare System, University of California San Diego, La Jolla, CA 92093, USA

**Keywords:** posttraumatic stress, PTSD, health related quality of life (HRQOL), neuroticism polygenic risk score, trauma, aging

## Abstract

Trauma-exposed adults with high levels of posttraumatic stress symptoms (PTSS) report poorer health-related quality of life (HRQOL), but less is known about the persistence of this relationship over time. Participants from the Vietnam Era Twin Study of Aging reported on PTSS, health, and sociodemographic characteristics at average age 38; 775 participants reported having been exposed to trauma. Later, at average ages 56 and 62, mental and physical HRQOL were assessed with the Short-Form 36. Premorbid risk for anxiety/neuroticism was evaluated with a polygenic risk score derived from a large genome-wide association study meta-analysis. In multivariate mixed models, having higher levels of PTSS, poorer self-rated health, lower income, and less education at age 38 were associated with worse physical and mental HRQOL two decades later. Chronic health problems at age 38 predicted midlife physical but not mental HRQOL. Although genetic risk for neuroticism was correlated with HRQOL and PTSS, it was no longer significant in multivariate models. Health-related quality of life (HRQOL) predicts morbidity and mortality independently of objective health measures; early interventions may help to mitigate the ongoing impact of trauma on quality of life.

## 1. Introduction

Health-related quality of life (HRQOL) has emerged as an important concept in aging research because it predicts mortality and morbidity above and beyond measures of objective health [1,2,3,4]. Measures of HRQOL typically encompass multiple components of subjective health such as the perceived severity and persistence of health problems, as well as their interference with life and their effect on mental wellbeing—none of which is captured by simply knowing if someone has a disease or disorder [2]. Adults with high levels of posttraumatic stress symptoms (PTSS) report poorer HRQOL, but less is known about the persistence of this relationship over time [5].

Military veterans, in particular, constitute a substantial cohort of aging adults that is more likely than adults in the general population to experience trauma [6]. Both veterans’ increased exposure to trauma as part of their military experience and their greater risk for childhood adversity puts them at higher risk for posttraumatic stress disorder (PTSD) than the general population [7,8]. However, despite being at greater risk for PTSD, veterans remain understudied in aging and HRQOL research [9]. Moreover, HRQOL in veterans is complex—in part because veterans enter the military in better health than their peers [10], but also because military service may provide an escape from a stressful childhood or young adult environment [10,11]. Military service may also provide “second chance” resources and opportunities otherwise not available, such as education, training, health care, housing, and food [12,13]. Reflecting this complexity, studies of trajectories of a single self-rated health item commonly used to assess HRQOL showed that veterans reported higher self-rated health than non-veterans at around age 50 but after age 70 their self-rated health was poorer and declined substantially faster than the non-veterans’ [14,15]. Differences within veterans also shifted over time [15], depending on factors such as age, war service, and experience. When combat veterans were compared with non-combat veterans, older combat veterans (age range 50–95) had poorer self-rated health, greater stress and distress than non-combat veterans [10]. Among middle-aged female veterans, recalled wartime stress exposures were associated with worse health functioning, and concurrent PTSD was associated with both poorer mental and physical QOL [16].

Some researchers have proposed that PTSD, even when symptoms are below the threshold for diagnosis, may be associated with more rapid aging [17,18]. Veterans with PTSD report poorer HRQOL overall [19] and poorer mental HRQOL in particular [20,21]. Vietnam war veterans with PTSD were found to have poorer physical health and report feeling more physical limitations than veterans without PTSD approximately 10 years after the end of the Vietnam war [22] and even 14 years later among those with persistent PTSD [23]. At midlife, veterans with PTSD reported worse HRQOL and higher levels of disability than those without PTSD [24]. These associations have been found even in individuals with symptoms not meeting diagnostic criteria for PTSD [25,26,27,28].

Inferences about associations between trauma exposure—an explicit diagnostic criterion for PTSD (APA, 2013)—and HRQOL have been limited by lack of information about an individual prior to trauma exposure. Anxiety, PTSS, PTSD, and HRQOL are each known to be influenced by both genetics and experience [29,30,31,32,33,34,35,36,37]. Studies find overlapping genetic influences between measures of anxiety, psychiatric disorders and HRQOL phenotypes [31,32,33]. Genetic influences account for approximately 17–33% of the variance depending on the HRQOL measure [1,36,38]—comparable to genetic influences on most personality traits—and are moderated by age, gender, and circumstances [1,29,39]. Individuals with higher genetic load for anxiety and neuroticism may struggle more to modulate emotional reactions and may experience stronger negative emotions like anxiety and irritability at lower levels of exposure than those with higher activation thresholds—predisposing them towards personality traits such as neuroticism, more physical and mental health disorders, and poorer HRQOL [40]. Genome-wide association studies (GWAS) have identified multiple “neuroticism” susceptibility loci [31,33,41]. Polygenic risk scores (PRS) can be used to summarize GWAS data into variables representing an individual’s genetic propensity for a phenotype such as anxiety proneness/neuroticism [31,32]. Few studies, however, have incorporated measures of genetic vulnerability into their exploration of associations between trauma exposure, PTSD symptoms, and HRQOL outcomes. In addition, there is some evidence that environmental influences may interact with genetic influences. Finkel et al., 2016, for instance, found that marriage appeared to be a protective factor for variations in self-rated HRQOL in men but not in women [39].

Most studies of HRQOL and response to trauma are cross-sectional or short-term, and focus on diagnosed PTSD rather than symptoms, unduly limiting attention to the impact of trauma exposure on the relatively few diagnosed individuals. Many existing studies of aging fail to inquire about trauma exposure or subsequent PTSD symptoms. In addition, although some researchers have proposed that PTSD and/or PTSS may be associated with more rapid pathological aging [17,18], no studies known to us have examined whether PTSS are associated with changes in HRQOL over time—a possible indicator of more rapid aging. Better knowledge about the persistence and extent of these associations can help inform health interventions for aging populations.

In this report we examine long-term associations between risk factors at mean age 38 and HRQOL approximately two decades later at mean ages 56 and 62. We predicted that development of PTSD symptoms by age 38 would be inversely associated with both physical and mental HRQOL (i.e., having more stress symptoms will be related to worse HRQOL outcomes). In order to test the hypothesis that PTSS are associated with signs of more rapid aging, we examined a PTSS by time interaction, wherein a greater decline in HRQOL over time among those with more PTSD symptoms would be one indication of more pathological aging. Finally, we explored the extent to which premorbid vulnerability for neuroticism modulated these associations. We predicted that those with higher genetic liability for neuroticism would report more PTSS and worse HRQOL.

## 2. Materials and Methods

### 2.1. Participants

The Vietnam Era Twin Study of Aging (VETSA) is a longitudinal study of risk and protective factors in aging [42,43]. The sample comprises community-dwelling, non-patient male twins who both served in the military at some time between 1965 and 1975. VETSA 1 participants (*n* = 1237; 2002–2008; mean age = 56, SD = 2.4; range 51–60) were randomly recruited from 3322 twin pairs from the nationally representative Vietnam Era Twin Registry (VETR) [44]. For inclusion in VETSA, participants were between 51 to 59 years old at recruitment and both members of the twin pair agreed to participate. Health and lifestyle characteristics of the VETSA sample are comparable to those of similarly aged men in the general population of the United States [45]. VETSA 1 participants were followed-up approximately 6 years later (2008–2013; VETSA 2) at mean age = 62, SD = 2.4; range 56–66, with 82% return rate. More details of the VETSA methods are published elsewhere [43,46].

### 2.2. Data Collection

Data for these analyses were collected in three waves: Survey of Health (SOH) in 1986–1987; VETSA 1 in 2003–2008; and VETSA 2 in 2009–2013. Previously collected SOH data on these participants were archived at the VETR and made available to the VETSA investigators. For the SOH data collection, participants completed a brief mailed questionnaire. At the time of the SOH assessment, participants were, on average, age 38 (SD = 2.5; range 32–42) [44].

In VETSA 1 and 2, participants were mailed a questionnaire booklet about a month prior to an in-person assessment to which they brought the completed questionnaires. During the in-person assessment they provided a blood specimen that was stored and later genotyped (see measures section). Henceforth, results from the three assessment waves are labeled age 38, age 56, and age 62, reflecting the mean age of participants at each assessment.

The research involved human participants and Institutional Review Board approval was obtained at all sites. The study was performed in accordance with the ethical standards as laid down in the 1964 Declaration of Helsinki and its later amendments or comparable ethical standards. The participants provided written consent prior to participation.

### 2.3. Measures: Age 38

Data collected at age 38 included measures of self-reported, self-rated health; posttraumatic stress symptoms; combat exposure; marital status; chronic health problems; income; alcohol consumption; and smoking status. Self-rated health was evaluated with the question “In general, how would you rate your health?” Scores ranged from 1 = excellent to 5 = poor (see Table 1). PTSD symptoms were assessed with a 13-item self-report PTSD questionnaire (“VETR-PTSD”) based on the Diagnostic and Statistical Manual of Mental Disorders, 3rd Edition Revised, the edition at the time [34,47,48]. Participants endorsed how frequently a symptom occurred in the past 6 months (very often, often, sometimes, almost never, or never). The VETR-PTSD measure has high reliability (α = 0.96) and validity. It correlates highly (r = 90) with the PTSD Checklist civilian version [PCL; [49]] when both instruments are administered simultaneously [24,34,50]. The self-report PTSD measure does not link symptoms to a particular life event.

Age 38 marital status was coded as: never married; married once; married multiple times. The chronic health index summed the presence of self-reported diabetes, chronic respiratory conditions, cancer, joint or skeletal disorders (i.e., osteoarthritis, rheumatoid arthritis), heart trouble, hypertension, and cirrhosis [51]. Household income during the past year was coded 1 (<$5000) to 10 (≥$50,000). Alcohol consumption in the past 2 weeks was coded as none/light (<1 drink per day)/moderate (<2 drinks per day)/heavy (>2 drinks per day). Cigarette smoking was coded as never/former/current. Participants also completed an 18-item questionnaire on types of combat-related experiences (yes/no). Scores were summed to create a combat exposure scale and validated against military records [52,53]; 51% had no combat exposures; 28% had 1–4 exposures; 16% had 5–9 exposures; and 4% had 10 or more.

### 2.4. Measures: Age 56 and 62

At ages 56 and 62, participants completed the Medical Outcomes Study Short-Form 36 version 1 (SF36), which is a widely used, reliable and valid measure of HRQOL [54]. The SF36 yields four physical subscales, four mental subscales, and two summary component scores. The four physical subscales evaluate limitations in physical mobility (Physical-Functioning), problems performing activities due to physical health problems (Role-Physical), limitations in activities due to pain (Bodily-Pain), and perceptions of general health (General-Health). The four mental subscales evaluate feelings of tiredness and low energy (Vitality), disengagement in social activities because of emotional problems (Social-Functioning), limitations with activities because of emotional problems (Role-Emotional), and feelings of unhappiness and depression (Mental health/Emotional Well-being). All subscales were standardized (mean = 0, standard deviation = 1); then, the four physical scales were averaged to create the Physical Component Summary score (Physical HRQOL) and the four mental scales averaged to create the Mental Component Summary score (Mental HRQOL). Higher scores indicate better HRQOL.

Other measures assessed at age 56 and 62 include lifetime education and age. Lifetime education was coded as years of formal education completed (0 = none to 20 = PhD, MD). At age 62, severity of eight different types of childhood traumas was assessed with the Pennebaker Childhood Trauma Scale [55,56]. For each item, a childhood trauma was coded as severe if it was rated six or seven on a 1–7 scale (1 = not traumatic to 7 = extremely traumatic); scores were then summed across the eight events. Participants reported zero to eight types of severe childhood experience: 210 (26.9%) reported no trauma; 401 (51.9%) reported 1–2 traumas; and 159 (22.2%) reported three or more.

### 2.5. Genotyping, Cleaning, and Imputation

A detailed description of VETSA genotyping procedures is available in Logue et al. [57]. In brief, we conducted genome-wide genotyping on individual dizygotic and unpaired twins and one randomly selected twin from each monozygotic pair using blood samples. Whole-genome genetic variation was assessed at deCODE Genetics (Reykjavík, Iceland) using Illumina HumanOmniExpress-24 v1.0A BeadChips (Illumina, San Diego, CA, USA). Before PRS calculations, we cleaned and conducted quality control of genotype data using PLINK v1.9 (Purcell Lab, Boston, MA, USA) [58]. Single nucleotide polymorphism weights (SNP weights) and principal components computed using PLINK v1.9 in conjunction with 1000 Genomes Phase 3 reference data [59] were used to identify a European ancestry subset of the data [60].

Principal components were computed based on a linkage-disequilibrium pruned set of 100,000 common (minor allele frequency > 0.05) genotyped SNPs. Within the European ancestry participants, principal components were recomputed for use as covariates for population substructure. Imputation of European-ancestry VETSA participants was performed in MiniMac on the Michigan Imputation Server at the University of Michigan, Ann Arbor, Michigan, USA. Using the 1000 Genomes phase 3 EUR (European descent) data as the haplotype reference panel. Before imputation, SNPs with more than 5% missing data or SNPs with Hardy–Weinberg equilibrium *p*-values < 10 ^−6^ were excluded. Only one twin per monozygotic twin pair was imputed and the results were applied to the un-genotyped co-twin.

### 2.6. Neuroticism/Anxiety Polygenic Propensity Score

A neuroticism polygenic risk score (neuroticism PRS) was created using summary data from a neuroticism GWAS conducted by Luciano et al. [32]. The neuroticism PRS at different threshold were computed using Plink 1.9 [58]. Following Luciano et al. (2018) [32], we examined *p*-value thresholds at *p* = 0.01, 0.05, 0.1, and 0.5. Single nucleotide polymorphisms (SNPs) for PRS formation were selected based on the SNPs in the VETSA genotyped sample that achieved an information threshold of 0.8 or higher in imputation and minor allele frequencies >1% in common with the Luciano et al. 2018 discovery SNP set and a reference data set to evaluate linkage disequilibrium patterns (1000G Phase 3 EUR). Subsequently, we performed clumping in Plink 1.9 using an r^2^ threshold of 0.1 in a 1000 kb window. Luciano et al. (2018) reported that the PRS threshold at *p* = 0.05 had the best signal-to-noise ratio so that threshold was used in further analyses.

Indeed, while all four PRS thresholds were significantly associated with the neuroticism phenotype in VETSA, none were stronger than the *p* = 0.05 threshold. Specifically, we validated the neuroticism PRS in the VETSA sample by examining associations between the neuroticism PRS and trait neuroticism phenotypes (i.e., the Multidimensional Personality Questionnaire (MPQ) negative emotionality score and the stress reaction subscale [61]) collected in VETSA 1. These MPQ scales represent reliable and valid measures of neuroticism/negative emotionality (see, for instance, Luciano et al. 2018 reproduction analyses). VETSA used a shortened (211 item) version of the MPQ [62,63]. Genetic propensity for neuroticism at *p* = 0.05 was significantly associated with trait negative emotionality (partial r = 0.16; *p* < 0.001) and stress reaction (partial r = 0.20; *p* < 0.001). Any analyses using the neuroticism PRS are adjusted for the first three principal components (PC) computed within European subgroups in order to adjust for cryptic population substructure (i.e., ancestry).

### 2.7. Final Analytic Sample

Only participants who had been trauma-exposed prior to the age 38 assessment were included in analyses since trauma exposure is a necessary diagnostic criterion for PTSD [64]. Determination of trauma exposure was based on: a) presence of a traumatic experience coded in the Diagnostic Interview Schedule Version III revised conducted in 1990 [37,65] and/or b) reporting at least one severe childhood trauma or combat exposure. The final analytic sample comprises the trauma-exposed participants who also had age 38 PTSS measures: *n* = 775 [48].

### 2.8. Statistical Analysis

All HRQOL measures were standardized before analyses (mean = 0, standard deviation = 1). We first used the weighted generalized estimating equations (WGEE) approach to model the relationship of PTSS with HRQOL outcomes at ages 56 and ages 62 by accounting for correlated outcomes in longitudinal assessments. WGEE addresses data clustering using a set of estimating equations rather than using random effects at model specification as in mixed-effects models [66]. Unlike the parametric generalized linear mixed-effects models, an alternative to modeling multi-level clustered data, the semi-parametric WGEE assumes no mathematical distributions such as normality and thereby provides valid inference for a broader class of data distributions under the missing at random mechanism [67]. WGEE also checks the missing completely at random (MCAR) assumption and reduces to the simpler generalized estimating equations (GEE) if MCAR is deemed appropriate. *p*-values for the missing data module were all greater than 0.05 for all outcomes in the analyses, suggesting that the assumption of MCAR was appropriate. Thus, we report the non-weighted generalized estimating equations (GEE) results for the final models.

The GEE removes the component for modeling the missing data mechanism in the WGEE, provides improved efficiency and fits a marginal model to longitudinal data. Regression parameters in the marginal model are interpreted as population-averaged. The correlations between twins are taken into account in the working matrix, and the time effects (repeated measures at the age 56 and age 62 assessments) are estimated as a fixed effect in the output. W(GEE) uses the sandwich variance estimate to account for correlated outcomes. The working correlation has the potential to increase inference efficiency, and WGEE provides valid inference regardless of how the working correlation is selected. The working independent correlation structure was used in all analyses in this study to ensure consistent estimates, as some covariates are time-varying [67].

We used SAS 9.4 for data analyses. Although the participants are twins, these are non-twin analyses and examine the role of genetics through the lens of a polygenic risk score based on GWAS. Because these analyses treat the twins as individuals, GEE models control for the non-independence of the twins and addresses nested correlations (i.e., twins and age) using sandwich variance estimates. Preliminary analyses examine associations between the neuroticism PRS and other measures, adjusting for the first three principal components reflecting ancestry. In order to test the hypotheses, we separately fit a GEE model for the overall physical and mental HRQOL scores. Models included: age 38 PTSS, time (categorical) as a fixed effect, the PTSS-by-time interaction, the neuroticism PRS, age 38 marital status, self-rated health, number of chronic health problems, income, alcohol consumption, smoking, number of combat and childhood traumas, and controlling for education and age at baseline. The PTSS-by time interaction allows us to see if symptoms have differential effects for the outcome at age 56 and age 62.

All statistical tests and confidence intervals are based on a two-tailed alpha = 0.05. Results for the eight SF36 subscales are provided in the Supplementary Tables S1–S3. Analyses of the eight subscales adjust for multiple comparisons using Bonferroni adjustments (alpha = 0.05/8 = 0.0063). This provides a conservative estimate since the SF36 subscale measures are highly correlated (i.e., subscale correlations range from r = 0.32 to r = 0.67; all *p* < 0.001).

## 3. Results

### 3.1. Descriptive Statistics/Preliminary Analyses

Participants were inducted into the military when they were, on average, 19.3 (SD 1.4) years old. The SOH assessment occurred approximately 18.6 years later when participants were, on average, 38 years old. By age 38, most participants had been married at least one time (91%). Median income was $30–34,000, which is roughly comparable to the median income ($32,787 in 1986) for United States men in this age group [68]. Mean education was 13.7 (SD 2.0, range 6–20) completed years, representing approximately 2 years additional education or training beyond high school. Descriptive statistics are provided in Table 1.

The neuroticism PRS was significantly, albeit weakly, correlated with age 38 PTSD symptoms (partial r = 0.10; *p* = 0.007 adjusted for three ancestry PCs), but not with any other age 38 measure (see Supplementary Table S1). The neuroticism PRS was significantly associated with Mental HRQOL (partial r = −0.13, *p* = 0.0003; r = −0.15, *p* < 0.0001 at ages 56 and 62 respectively). The associations between the neuroticism PRS and Physical HRQOL were partial r = −0.05, *p* = 0.20; and partial r = −0.08, *p* = 0.05; age 56 and 62 respectively. Thus, higher genetic propensity for neuroticism was associated with worse HRQOL.

### 3.2. Longitudinal Multivariate GEE Models

Using longitudinal multivariate GEE models, we examined the extent to which the age 38 measures predict Physical and Mental HRQOL at midlife (Table 2). In multivariate models, there were significant effects of PTSS, self-rated health, income, lifetime education, and childhood trauma on later Physical and Mental HRQOL (shown are Type 3 effects). Having more posttraumatic stress symptoms, worse self-rated health, lower education and income, and more severe childhood traumas were each associated with worse HRQOL outcomes. The number of chronic health problems at age 38 significantly predicted later Physical HRQOL but not Mental HRQOL. The effects of the time, the time-by-PTSS interaction, combat exposure, and neuroticism PRS were non-significant. The lack of a time-by-PTSS interaction indicates that PTSS had the same effect at age 56 and 62. Results for the separate SF36 HRQOL subscales are provided in Supplementary Tables S2 and S3. Results are generally consistent with the overall component scores.

## 4. Discussion

In a sample of men who had all been exposed to trauma, multiple potentially modifiable risk factors were associated with their midlife physical and mental quality of life across more than two decades. As predicted, participants with more symptoms of posttraumatic stress at age 38 had worse HRQOL in midlife. We did not find support for the hypothesis that PTSD symptoms would be associated with more rapid aging, as shown by the non-significant PTSS by time interaction. Rather, across the 6 years of the midlife follow-up, mental and physical HRQOL were highly correlated and showed little change. Thus, while participants with more PTSD symptoms had poorer HRQOL overall, they did not decline at a different rate than those with lower symptoms within this 6-year timeframe. In partial support of our hypothesis about genetic influences, genetic propensity for neuroticism was associated with both age 38 PTSD symptoms and with midlife mental and physical HRQOL, albeit weakly, in models adjusted only for ancestry. In multivariate models, however, those associations were no longer significant. This is due, in part, to the fact that the genetic influences were associated with both the predictor variable (PTSD symptoms) as well as the HRQOL outcome measures. In addition, associations between the neuroticism polygenic score and these measures were small, consistent with previous research on the PRS [32]. It may be that other influences—possibly life experiences or personal characteristics—as yet to be determined, mediate or moderate those associations. In summary, developing PTSD symptoms in response to trauma put individuals at greater risk for worse quality of life outcomes decades later, which may ultimately contribute to increased risk for disease and mortality as they age [3,4,69]. We found, for instance, that PTSD symptoms from age 38 were associated with structural deficits in brain regions regulating stress responsivity and adaptation at midlife [70,71].

Multiple other age 38 modifiable risk factors were associated with midlife HRQOL in the multivariate models, including self-rated health, education, income, and childhood traumas. Although chronic illnesses were infrequent at age 38, having more chronic illness at this early age was associated with poorer midlife physical but not mental HRQOL. Because of the low prevalence of health problems at age 38, we did not anticipate an association between perceptions of health at age 38 and later HRQOL. It appears that an individual’s health perceptions have long-term implications, separately from the presence of chronic illnesses. Self-rated health measures may capture trait-like evaluations of health [2] in part accounted for by genetic influences [1,38].

This study of a community-dwelling, non-patient sample of trauma-exposed veterans is unique in the strength and duration of its longitudinal data, as well as in its use of a neuroticism polygenic risk score as a measure of one type of pre-trauma liability. To our knowledge, this is the only longitudinal study to date that incorporates genetic risks into models predicting HRQOL. The longitudinal relationships between PTSS and HRQOL are consistent with those from cross-sectional and short-term findings on subthreshold PTSD [25,26]. Mota et al. also found that PTSD symptoms may reemerge or occur with late-onset in older veterans and are likely exacerbated by earlier executive dysfunction, which may restrict flexible coping responses as new stressors appear in late life [72]. Thus, there may be value in clinicians evaluating symptomatic responses to trauma whether or not responses meet diagnostic criteria [73]. These long-term influences on later HRQOL suggest multiple possibly modifiable sources of risk and protection from relatively early in adulthood.

Multiple mechanisms have been proposed that link trauma and PTSD symptoms with later life HRQOL. It may be that premorbid characteristics are associated with tendencies to interpret, react to, or cope with stressful stimuli in particular ways [74]. Some researchers theorize that aberrant threat-related processes may partly underlie heightened and prolonged reactivity to a stressful event [75]. Stress and stress reactivity, for instance, increase allostatic load, contributing to changes in physiological systems [76,77,78,79], which then may also affect perceptions of health. Trauma and PTSD symptoms contribute directly and indirectly to stress hormone dysregulation and related outcomes [80,81,82,83,84]. Stress mediators such as corticotropin releasing factor are implicated in anxiety and stress disorders and are moderately heritable [84,85,86,87]. Additionally, there is evidence that stress responses are associated with brain regions that play important roles in how events are perceived, experienced, interpreted, and managed [70,88,89]. Moreover, emerging evidence suggests genetic variation may increase risk for anxiety disorders and affect brain neurocircuitry [90]. Personality characteristics, which are also genetically mediated, may underlie self-reporting of characteristics such as PTSD symptoms and HRQOL. In these analyses, however, genetic influences did not contribute meaningfully to the HRQOL outcomes.

Being able to examine pre-morbid characteristics (i.e., by using the neuroticism PRS) is important because prior risk is typically difficult to account for in most studies of trauma [40]. In this study, genetic vulnerability for neuroticism correlated modestly with multiple self-report measures (e.g., PTSS, HRQOL measures), indicating that genetic vulnerability underlies and is common to these subjective indicators. It was not associated with reports of combat exposures, childhood traumas, income or education. Some have suggested personalized medicine should be more attentive to genetic liability for neuroticism [91] because of the high emotional and financial costs associated with mental and physical health outcomes. The small associations found here, however, suggest other influences likely modulate these associations and there are likely better targets for intervention.

The present study has several limitations. The sample is all men due to the original ascertainment of the sample as twin veterans who both served in the military between 1965 and 1975 when there were few women serving (especially female twin pairs). In addition, the current state of research using the neuroticism PRS limits the sample to those of Caucasian/European heritage. Thus, we cannot generalize across sex or ethnicity. Some studies have found sex differences in HRQOL outcomes [20,21]. The sample comprises veterans; as a subgroup, veterans are at higher risk for traumatic exposures. Veteran status, however, is seldom considered in many of our existing studies of aging. Although most measures used as predictors or covariates were assessed when the men were in their 30s, the childhood trauma data were collected at age 62 and thus may be subject to recall bias. Finally, the weaker associations with the specific SF36 subscales is likely due, in part, to lower reliability for those subscales, given that the subscales have fewer items compared with the overall score. In addition, we do not know about neurotoxin exposure during military service which may have effects on both health and HRQOL. Limitations of the study are offset by its strengths which include an assessment of PTSS and covariates relatively early in adulthood, use of GWAS data to create a premorbid risk indicator, and longitudinal measures of HRQOL, all in a community-based, richly characterized, non-patient sample.

## 5. Conclusions

Better HRQOL is linked to health, good immune function, happiness, longevity, and lower health care costs in aging adults [92,93]. Health care costs have been found to be significantly higher for adults with posttraumatic stress symptom scores on the PCL in the clinically meaningful range (PCL > 44) or moderate range (PCL 30–44) compared with those with a low number of symptoms [94]. Thus, our results showing that having more symptoms of posttraumatic stress is associated with poorer mental and physical quality of life over two decades, have meaningful clinical, public health, social, and socioeconomic implications for the care of our growing population of older adults [72,94,95]. Identification of environmental influences is important and could potentially lead to the development of interventions that may then be instrumental in improving health outcomes.

## Figures and Tables

**Table 1:** Descriptive Statistics.

Measure	Survey of Health	VETSA 1	VETSA 2
Age: mean SD	37.9 (2.5)	56.0 (2.4)	62.0 (2.4)
# Chronic Health Problems: mean SD	0.36 (0.67) range 0–5	1.15 (1.24) range 0–9	1.71 (1.47) range 0–15
Self-rated health			
Excellent	321 (35.08%)	77 (9.59%)	58 (7.45%)
Very Good	397 (43.39%)	287 (35.74%)	279 (35.82%)
Good	168 (18.36%)	328 (40.85%)	314 (40.31%)
Fair	27 (2.95%)	98 (12.20%)	103 (13.22%)
Poor	2 (0.22%)	13 (1.62%)	25 (3.21%)
SF36 Subscales			
Bodily Pain		9.01 (2.27)	8.61 (2.27)
General Health		18.76 (4.17)	18.21 (4.50)
Physical Functioning		26.51 (3.98)	25.31 (4.72)
Role Physical		7.24 (1.37)	6.99 (1.51)
Role Emotional		5.60 (0.88)	5.53 (0.97)
Mental Health		23.99 (4.32)	24.46 (4.40)
Vitality		16.14 (3.99)	16.02 (4.12)
Social Functioning		8.83 (1.74)	8.69 (1.86)

The age 38 self-rated health item was significantly associated with age 56 HRQOL (r = −0.36; r = −0.25 *p* < 0.0001, for Physical and Mental HRQOL respectively), with similar associations at age 62 (r = −0.31; r = −0.25 *p* < 0.0001, for Physical and Mental HRQOL respectively) indicating moderate stability of quality of life across more than two decades. Physical and Mental HRQOL measures were significantly correlated at ages 56 and 62. Mental HRQOL at age 56 correlated r = 0.66 with Mental HRQOL at age 62; Physical HRQOL at age 56 correlated r = 0.71 (*p* < 0.0001) with Physical HRQOL at age 62. Cross-correlations between Physical and Mental HRQOL were also high (r = 0.52 to 0.54; *p* < 0.0001) over time and within time (r = 0.69 at age 56 and r = 0.73 at age 62, *p* < 0.0001).

**Table 2:** Age 38 Characteristics and Physical and Mental HRQOL Component Scores at Midlife in Trauma-Exposed Men (Type 3 tests of fixed effects).

Measure	Physical Health Component		Mental Health Component	
	Estimate (SE)	95% CI	Estimate (SE)	95% CI
Intercept	−0.65 (0.61)	[−1.85, 0.55]	−0.76 (0.63)	[−2.00, 0.48]
PTSS	**−0.02 (0.01)**	**[−0.04, −0.01]**	**−** **0.04 (0.01)**	**[−** **0.05, −** **0.02]**
Time (age 56, age 62)	0.04 (0.09)	[−0.13, 0.21]	0.07 (0.09)	[−0.12, 0.25]
PTSS * Time	−0.00 (0.00)	[−0.01, 0.01]	−0.00 (0.00)	[−0.01 ,0.01]
Neuroticism PRS	−0.11 (0.09)	[−0.29, 0.07]	−0.15 (0.10)	[−0.34, 0.04]
Age at SOH	0.02 (0.01)	[−0.00, 0.05]	**0.03 (0.01)**	**[0.01, 0.06]**
Education	**0.05 (0.01)**	**[0.02, 0.08]**	**0.03 (0.01)**	**[0.00, 0.06]**
Combat exposure	−0.01 (0.01)	[−0.03, 0.02]	−0.00 (0.01)	[−0.03, 0.02]
Childhood trauma	**−0.06 (0.02)**	**[−0.10, −0.01]**	**−** **0.06 (0.02)**	**[−** **0.10, −** **0.01]**
Self-rated health	**−0.21 (0.04)**	**[−0.29, −0.13]**	**−** **0.12 (0.04)**	**[−** **0.19, −** **0.05]**
Married once	−0.11 (0.12)	[−0.34, 0.13]	0.08 (0.14)	[−0.18, 0.35]
Married > once	−0.16 (0.13)	[−0.41, 0.09]	0.15 (0.14)	[−0.14, 0.43]
Income	**0.05 (0.02)**	**[0.02, 0.08]**	**0.04 (0.01)**	**[0.02, 0.07]**
Current smoker	−0.14 (0.08)	[−0.30, 0.02]	−0.09 (0.07)	[−0.23, 0.05]
Former smoker	−0.11 (0.06)	[−0.24, 0.01]	−0.12 (0.06)	[−0.24, 0.01]
Alcohol consumption	−0.00 (0.00)	[−0.01, 0.00]	−0.00 (0.00)	[−0.01, 0.00]
Chronic health problems	**−0.13 (0.06)**	**[−0.25, −0.01]**	−0.07 (0.05)	[−0.17, 0.04]
PTSS * Neuroticism PRS	0.00 (0.00)	[−0.00, 0.01]	0.00 (0.00)	[−0.01, 0.01]
PC1	1.18 (0.77)	[−0.32, 2.69]	0.82 (0.66)	[−0.47, 2.12]
PC2	−0.74 (0.84)	[−2.38, 0.90]	−0.40 (0.88)	[−2.12, 1.32]
PC3	**−2.35 (0.73)**	**[−3.78, −0.92]**	**−** **1.49 (0.66)**	**[−** **2.78, −** **0.20]**

Abbreviations: CI—Confidence Interval; PTSS—Posttraumatic Stress Symptoms; PRS—Polygenic Risk Score; PC (1–3)—first 3 principal components representing ancestry; SOH—Survey of Health (average age 38); * PTSS, age, combat exposure, self-rated health, marriage, income, smoking, alcohol consumption and health problems were assessed at Survey of Health. Time is a fixed effect and represents the assessment at age 56 (VETSA 1) and age 62 (VETSA 2) HRQOL assessments. Bolded items are significant at *p* < 0.05, two tailed.

## Supplementary Materials

The following are available online at https://www.mdpi.com/1660-4601/17/12/4554/s1, Table S1: Partial Correlations between Neuroticism Polygenic Risk Scores, Posttraumatic Stress Symptoms and Covariates, Table S2: Associations between Posttraumatic Stress Symptoms at Age 38 and Short Form 36 Mental HRQOL Subscales, Table S3: Associations between Posttraumatic Stress Symptoms at Age 38 and Short Form 36 Physical HRQOL Subscales.

## References

[1] Franz C.E., Finkel D., Panizzon M.S., Spoon K., Christensen K., Gatz M., Kremen W.S., Krueger R., Neiderhiser J., Reynolds C. (2017). Facets of subjective health from early adulthood to old age. J. Aging Health.

[2] Idler E., Cartwright K. (2018). What do we rate when we rate our health? Decomposing age-related contributions to self-rated health. J. Health Soc. Behav..

[3] Idler E., Benyamini Y. (1997). Self-rated health mortality: A review of twenty-seven community studies. J. Health Soc. Behav..

[4] Latham K., Peek C.W. (2013). Self-rated health morbidity onset among late midlife US adults. J. Gerontol. B Psychol. Sci. Soc. Sci..

[5] Byers A.L., Covinsky K.E., Neylan T.C., Yaffe K. (2014). Chronicity of posttraumatic stress disorder risk of disability in older persons. JAMA Psychiatry.

[6] US Department of Veterans Affairs, Office of the Actuary, Veteran Population Projection Model (VetPop) 2011 Department of Veterans Affairs Statistics at a Glance. https://www.va.gov/vetdata/docs/Quickfacts/Homepage_slideshow_FINAL.pdf.

[7] Cabrera O.A., Hoge C.W., Bliese P.D., Castro C.A., Messer S.C. (2007). Childhood adversity combat as predictors of depression post-traumatic stress in deployed troops. Am. J. Prev. Med..

[8] Stellman S., Stellman J., Koenen K. (2000). Enduring social behavioral effects of exposure to military combat in Vietnam. Ann. Epidemiol..

[9] Spiro A., Settersten R.A., Aldwin C.M. (2018). Long-term Outcomes of Military Service: The Health Well-Being of Aging Veteran.

[10] Aldwin C.M., Park C.L., Choun S., Lee H., Spiro. A., Settersten R.A., Aldwin C.M. (2018). The impact of military service on stress health, well-being in later life. Long-Term Outcomes of Military Service: The Health Well-Being of Aging Veterans.

[11] Kang S., Aldwin C.M., Choun S., Spiro A. (2016). A life-span perspective on combat exposure PTSD symptoms in later life: Findings from the VA normative aging study. Gerontologist.

[12] Werner E.E., Smith R.S. (2001). Journeys from Childhood to Midlife: Risk Resilience and Recovery.

[13] Werner E.E. (1993). Risk, resilience, recovery: Perspectives from the Kauai longitudinal study development. Psychopathology.

[14] Wilmoth J.M., London A.S., Oliver W.J., Spiro A., Settersten R.A., Aldwin C.M. (2018). Military Service Experiences Older Men’s Trajectories of Self-Rated Health. Long-Term Outomces of Military Sercice: The Health Well-Being of Aging Veterans.

[15] Wilmoth J.M., London A.S., Parker W.M. (2010). Military service men’s health trajectories in later life. J. Gerontol. B Psychol. Sci. Soc. Sci..

[16] Smith B.N., Spiro A., Frayne S.M., Kimerling R., Cypel Y., Reinhard M.J., Kilbourne A.M., Magruder K.M. (2020). Impact of wartime stress exposures mental health on later-life functioning disability in Vietnam-era women veterans: Findings from the health of Vietnam-era women’s study. Psychosom. Med..

[17] Roberts A.L., Koenen K.C., Chen Q., Gilsanz P., Mason S.M., Prescott J., Ratanatharathorn A., Rimm E.B., Sumner J.A., Winning A. (2017). Posttraumatic stress disorder accelerated aging: PTSD, leukocyte telomere length in a sample of civilian women. Depress. Anxiety.

[18] Lohr J.B., Palmer B.W., Eidt C.A., Aailaboyina S., Mausbach B.T., Wolkowitz O.M., Thorp S.R., Jeste D.V. (2015). Is post-traumatic stress disorder associated with premature senescence? A review of the literature. Am. J. Geriatr. Psychiatry.

[19] Schnurr P.P., Lunney C.A., Bovin M.J., Marx B.P. (2009). Posttraumatic stress disorder quality of life: Extension of findings to veterans of the wars in Iraq and Afghanistan. Clin. Psychol. Rev..

[20] Fang S.C., Schnurr P.P., Kulish A.L., Holowka D.W., Marx B.P., Keane T.M., Rosen R. (2015). Psychosocial functioning health-related quality of life associated with posttraumatic stress disorder in male and female Iraq and Afghanistan war veterans: The VALOR registry. J. Womens Health (Larchmt.).

[21] Vogt D., Smith B.N., Fox A.B., Amoroso T., Taverna E., Schnurr P.P. (2017). Consequences of PTSD for the work family quality of life of female male U.S. Afghanistan Iraq War veterans. Soc. Psychiatry Psychiatr. Epidemiol..

[22] Zatzick D.F., Marmar C.R., Weiss D.S., Browner W.S., Metzler T.J., Golding J.M., Stewart A., Schlenger W.E., Wells K.B. (1997). Posttraumatic stress disorder functioning quality of life outcomes in a nationally representative sample of male Vietnam veterans. Am. J. Psychiatry.

[23] Koenen K.C., Stellman S.D., Sommer J.F., Stellman J.M. (2008). Persisting posttraumatic stress disorder symptoms their relationship to functioning in Vietnam veterans: A 14-year follow-up. J. Trauma Stress.

[24] Goldberg J., Magruder K.M., Forsberg C.W., Kazis L.E., Ustun T.B., Friedman M.J., Litz B.T., Vaccarino V., Heagerty P.J., Gleason T.C. (2014). The association of PTSD with physical mental health functioning disability (VA Cooperative Study #569: The course consequences of posttraumatic stress disorder in Vietnam-era veteran twins). Qual. Life Res..

[25] Mota N.P., Tsai J., Sareen J., Marx B.P., Wisco B.E., Harpaz-Rotem I., Southwick S.M., Krystal J.H., Pietrzak R.H. (2016). High burden of subthreshold DSM-5 post-traumatic stress disorder in US military veterans. World Psychiatry.

[26] El-Gabalawy R., Blaney C., Tsai J., Sumner J.A., Pietrzak R.H. (2018). Physical health conditions associated with full subthreshold PTSD in US military veterans: Results from the National Health Resilience in Veterans Study. J. Affect. Disord..

[27] Pereira M.G., Machado J.C., Pereira M., Lopes C., Pedras S. (2019). Quality of life in elderly Portuguese war veterans with post-traumatic stress symptoms. Patient Relat. Outcome Meas..

[28] Pagotto L.F., Mendlowicz M.V., Coutinho E.S., Figueira I., Luz M.P., Araujo A.X., Berger W. (2015). The impact of posttraumatic symptoms comorbid mental disorders on the health-related quality of life in treatment-seeking PTSD patients. Compr. Psychiatry.

[29] Finkel D., Franz C.E., Christensen K., Reynolds C.A., Pedersen N.L. (2020). Longitudinal twin study of subjective health: Differences in genetic environmental components of variance across age sex. J. Gerontol. B Psychol. Sci. Soc. Sci..

[30] Harris S.E., Hagenaars S.P., Davies G., David Hill W., Liewald D.C.M., Ritchie S.J., Marioni R.E. (2017). METASTROKE Consortium; International Consortium for Blood Pressure Genome-Wide Association Studies; CHARGE Consortium Aging and Longevity Group; et al. Molecular genetic contributions to self-rated health. Int. J. Epidemiol..

[31] Lo M.T., Hinds D.A., Tung J.Y., Franz C., Fan C.C., Wang Y., Marioni R.E., METASTROKE Consortium International Consortium for Blood Pressure Genome-Wide Association Studies, International Consortium for Blood Pressure Genome-Wide Association Studies, CHARGE Consortium Aging and Longevity Group (2017). Genome-wide analyses for personality traits identify six genomic loci show correlations with psychiatric disorders. Nat. Genet..

[32] Luciano M., Hagenaars S.P., Davies G., Hill W.D., Clarke T.K., Shirali M., Harris S.E., Marioni R.E., Liewald D.C., Fawns-Ritchie C. (2018). Association analysis in over 329,000 individuals identifies 116 independent variants influencing neuroticism. Nat. Genet..

[33] Okbay A., Baselmans B.M., De Neve J.E., Turley P., Nivard M.G., Fontana M.A., Meddens S.F., Linnér R.K., Rietveld C.A., Derringer J. (2016). Genetic variants associated with subjective well-being depressive symptoms, neuroticism identified through genome-wide analyses. Nat. Genet..

[34] True W.R., Rice J., Eisen S.A., Heath A.C., Goldberg J., Lyons M.J., Nowak J. (1993). A twin study of genetic environmental contributions to liability for posttraumatic stress symptoms. Arch. Gen. Psychiatry.

[35] Zettergren A., Kern S., Ryden L., Ostling S., Blennow K., Zetterberg H., Falk H., Skoog I. (2018). Genetic variation in FOXO3 is associated with self-rated health in a population-based sample of older individuals. J. Gerontol. A Biol. Sci. Med. Sci..

[36] Romeis J.C., Heath A.C., Xian H., Eisen S.A., Scherrer J.F., Pedersen N.L., Fu Q., Bucholz K.K., Goldberg J., Lyons M.J. (2005). Heritability of SF-36 among middle-age middle-class male-male twins. Med. Care.

[37] Robins L.N., Helzer J.E., Cottler L., Goldring E. (1989). Diagnostic Interview Schedule (Version III Revised).

[38] Romeis J.C., Scherrer J.F., Xian H., Eisen S.A., Bucholz K., Heath A.C., Goldberg J., Lyons M.J., Henderson W.G., True W.R. (2000). Heritability of self-reported health. Health Serv. Res..

[39] Finkel D., Franz C.E., Horwitz B., Christensen K., Gatz M., Johnson W., Kaprio J., Korhonen T., Niederheiser J., Petersen I. (2016). Gender differences in marital status moderation of genetic environmental influences on subjective health. Behav. Genet..

[40] Eaves L.J., Eysenck H.J., Martin N.G. (1989). Genes Culture Personality: An Empirical Approach.

[41] Lo M.T., Wang Y., Kauppi K., Sanyal N., Fan C.C., Smeland O.B., Schork A., Holland D., Hinds D.A., Tung J.Y. (2017). Modeling prior information of common genetic variants improves gene discovery for neuroticism. Hum. Mol. Genet..

[42] Kremen W.S., Franz C.E., Lyons M.J. (2013). VETSA: The Vietnam Era Twin Study of Aging. Twin Res. Hum. Genet..

[43] Kremen W.S., Thompson-Brenner H., Leung Y.J., Grant M.D., Franz C.E., Eisen S.A., Jacobson K.C., Boake C., Lyons M.J. (2006). Genes environment, and time: The Vietnam Era Twin Study of Aging (VETSA). Twin Res. Hum. Genet..

[44] Eisen S.A., True W.R., Goldberg J., Henderson W., Robinette C.D. (1987). The Vietnam Era Twin (VET) Registry: Method of construction. Acta Genet. Med. Gemello.

[45] Centers for Disease Control Prevention (CDC) (2007). Health Data for all Ages.

[46] Franz C.E., Panizzon M.S., Eaves L.J., Thompson W., Lyons M.J., Jacobson K.C., Tsuang M., Glatt S.J., Kremen W.S. (2012). Genetic environmental multidimensionality of Well– and ILL–Being in middle aged twin men. Behav. Genet..

[47] Cooper A.M., Michels R. (1987). Diagnostic Statistical Manual of Mental Disorders, revised.

[48] Goldberg J., True W.R., Eisen S.A., Henderson W.G. (1990). A twin study of the effects of the Vietnam War on posttraumatic stress disorder. JAMA.

[49] Weathers F.W., Litz B.T., Herman D.S., Huska J.A., Keane T.M. The PTSD Checklist (PCL): Reliability validity, diagnostic utility. Proceedings of the Annual Convention of the International Society for Traumatic Stress Studies.

[50] Magruder K., Yeager D., Goldberg J., Forsberg C., Litz B., Vaccarino V., Friedman M., Gleason T., Huang G., Smith N. (2015). Diagnostic performance of the PTSD checklist the Vietnam era twin registry PTSD scale. Epidemiol. Psychiatr. Sci..

[51] Charlson M., Szatrowski T.P., Peterson J., Gold J. (1994). Validation of a combined comorbidity index. J. Clin. Epidemiol..

[52] Janes G.R., Goldberg J., Eisen S.A., True W.R. (1991). Reliability validity of a combat exposure index for Vietnam era veterans. J. Clin. Psychol..

[53] Lyons M.J., Goldberg J., Eisen S.A., True W., Tsuang M.T., Meyer J.M., Henderson W.G. (1993). Do genes influence exposure to trauma? A twin study of combat. Am. J. Med. Genet..

[54] Ware J.E., Sherbourne C.D. (1992). The MOS 36-item Short-Form Health Survey (SF-36): I Conceptual framework item selection. Med. Care.

[55] Pennebaker J.W. (1999). The effects of traumatic disclosure on physical mental health: The values of writing talking about upsetting events. Int. J. Emerg. Ment. Health.

[56] Pennebaker J.W., Susman J.R. (1988). Disclosure of traumas psychosomatic processes. Soc. Sci. Med..

[57] Logue M.W., Panizzon M.S., Elman J.A., Gillespie N.A., Hatton S.N., Gustavson D.E., Andreassen O.A., Dale A.M., Franz C.E., Lyons M.J. (2019). Use of an Alzheimer’s disease polygenic risk score to identify mild cognitive impairment in adults in their 50s. Mol. Psychiatry.

[58] Chang C.C., Chow C.C., Tellier L.C., Vattikuti S., Purcell S.M., Lee J.J. (2015). Second-generation PLINK: Rising to the challenge of larger richer datasets. Gigascience.

[59] Auton A., Brooks L.D., Durbin R.M., Garrison E.P., Kang H.M., Korbel J.O., Marchini J.L., McCarthy S., McVean G.A., 1000 Genomes Project Consortium (2015). A global reference for human genetic variation. Nature.

[60] Chen C.Y., Pollack S., Hunter D.J., Hirschhorn J.N., Kraft P., Price A.L. (2013). Improved ancestry inference using weights from external reference panels. Bioinformatics.

[61] Tellegen A., Lykken D.T., Bouchard T.J., Wilcox K.J., Segal N.L., Rich S. (1988). Personality similarity in twins reared apart together. J. Pers. Soc. Psychol..

[62] Krueger R.F., Caspi A., Moffitt T.E., Silva P.A., McGee R. (1996). Personality traits are differentially linked to mental disorders: A multitrait-multidiagnosis study of an adolescent birth cohort. J. Abnorm. Psychol..

[63] Franz C.E., York T.P., Eaves L.J., Prom-Wormley E., Jacobson K.C., Lyons M.J., Grant M.D., Xian H., Panizzon M.S., Jimenez E. (2011). Adult romantic attachment negative emotionality and depressive symptoms in middle aged men: A multivariate genetic analysis. Behav. Genet..

[64] American Psychiatric Association (2013). Diagnostic Statistical Manual of Mental Disorders.

[65] Tsuang M.T., Bar J.L., Harley R.M., Lyons M.J. (2001). The Harvard Twin Study of Substance Abuse: What we have learned. Harv. Rev. Psychiatry.

[66] Kowalski J., Tu X.M. (2007). Modern Applied U-Statistics.

[67] Tang W., He H., Tu X.M. (2012). Applied Categorical Count Data Analysis.

[68] Semega J., Kollar M., Creame J., Mohanty A., US Census Bureau (2019). Current Population Survey Annual Social Economic Supplements (Table H-10). Age of Householder: All Races by Median Mean Income: 1967 to 2017–2018.

[69] Idler E.L., Russell L.B., Davis D. (2000). Survival functional limitations, and self-rated health in the NHANESI epidemiologic follow-up study, 1992. First national health nutrition examination survey. Am. J. Epidemiol..

[70] Franz C.E., Hatton S.N., Hauger R.L., Kredlow M.A., Dale A.M., Eyler L., McEvoy L.K., Fennema-Notestine C., Hagler D., Jacobson K.C. (2019). Posttraumatic stress symptom persistence across 24 years: Association with brain structures. Brain Imaging Behav..

[71] Franz C.E., Lyons M.J., Kremen W.S., Spiro A., Settersten R.A., Aldwin C.M. (2018). Long-term influences of combat exposure post-traumatic stress symptoms on brain structure health, and functioning: The Vietnam era twin study of aging. Long-Term Outcomes of Military Service: The Health Wellbeing of Aging Veterans.

[72] Mota N.P., Tsai J., Kirwin P.D., Harpaz-Rotem I., Krystal J.H., Southwick S.M., Pietrzak R.H. (2016). Late-life exacerbation of PTSD symptoms in US veterans: Results from the National Health Resilience in Veterans Study. J. Clin. Psychiatry.

[73] Sheikh M.A., Abelsen B., Olsen J.A. (2016). Clarifying associations between childhood adversity social support behavioral factors and mental health, health and well-being in adulthood: A population-based study. Front. Psychol..

[74] Daviu N., Bruchas M.R., Moghaddam B., Sandi C., Beyeler A. (2019). Neurobiological links between stress anxiety. Neurobiol. Stress.

[75] Rabellino D., Densmore M., Frewen P.A., Theberge J., Lanius R.A. (2016). The innate alarm circuit in post-traumatic stress disorder: Conscious subconscious processing of fear-, trauma-related cues. Psychiatry Res. Neuroimaging.

[76] Kremen W.S., O’Brien R.C., Panizzon M.S., Prom-Wormley E., Eaves L.J., Eisen S.A., Eyler L.T., Hauger R.L., Fennema-Notestine C., Fischl B. (2010). Salivary cortisol prefrontal cortical thickness in middle-aged men: A twin study. Neuroimage.

[77] Miller G.E., Chen E., Fok A.K., Walker H., Lim A., Nicholls E.F., Cole S., Kobor M.S. (2009). Low early-life social class leaves a biological residue manifested by decreased glucocorticoid increased proinflammatory signaling. Proc. Natl. Acad. Sci. USA.

[78] McGowan P.O., Sasaki A., D’Alessio A.C., Dymov S., Labonte B., Szyf M., Turecki G., Meaney M.J. (2009). Epigenetic regulation of the glucocorticoid receptor in human brain associates with childhood abuse. Nat Neurosci..

[79] McEwen B.S., Bowles N.P., Gray J.D., Hill M.N., Hunter R.G., Karatsoreos I.N., Nasca C. (2015). Mechanisms of stress in the brain. Nat. Neurosci..

[80] Engert V., Buss C., Khalili-Mahani N., Wadiwalla M., Dedovic K., Pruessner J.C. (2010). Investigating the association between early life parental care stress responsivity in adulthood. Dev. Neuropsychol..

[81] Heim C., Newport D.J., Mletzko T., Miller A.H., Nemeroff C.B. (2008). The link between childhood trauma depression: Insights from HPA axis studies in humans. Psychoneuroendocrinology.

[82] Saridjan N.S., Huizink A.C., Koetsier J.A., Jaddoe V.W., Mackenbach J.P., Hofman A., Kirschbaum C., Verhulst F.C., Tiemeier H. (2010). Do social disadvantage early family adversity affect the diurnal cortisol rhythm in infants? The Generation R Study. Horm. Behav..

[83] Davidson R.J., McEwen B.S. (2012). Social influences on neuroplasticity: Stress interventions to promote well-being. Nat. Neurosci..

[84] Futch H.S., Croft C.L., Truong V.Q., Krause E.G., Golde T.E. (2017). Targeting psychologic stress signaling pathways in Alzheimer’s disease. Mol. Neurodegener..

[85] McEwen B.S., Nasca C., Gray J.D. (2016). Stress effects on neuronal structure: Hippocampus amygdala and prefrontal cortex. Neuropsychopharmacology.

[86] Hauger R.L., Risbrough V., Oakley R.H., Olivares-Reyes J.A., Dautzenberg F.M. (2009). Role of CRF receptor signaling in stress vulnerability anxiety and depression. Ann. N. Y. Acad. Sci..

[87] Arnsten A.F. (2015). Stress weakens prefrontal networks: Molecular insults to higher cognition. Nat. Neurosci..

[88] Sun D., Peverill M.R., Swanson C.S., McLaughlin K.A., Morey R.A. (2018). Structural covariance network centrality in maltreated youth with posttraumatic stress disorder. J. Psychiatr. Res..

[89] Logue M.W., van Rooij S.J.H., Dennis E.L., Davis S.L., Hayes J.P., Stevens J.S., Densmore M., Haswell C.C., Ipser J., Koch S. (2018). Smaller hippocampal volume in posttraumatic stress disorder: A multisite ENIGMA-PGC study: Subcortical volumetry results from posttraumatic stress disorder consortia. Biol. Psychiatry..

[90] van der Merwe C., Jahanshad N., Cheung J.W., Mufford M., Groenewold N.A., Koen N., Ramesar R., Dalvie S., Knowles J.A., ENIGMA Consortium PGC-PTSD (2019). Concordance of genetic variation that increases risk for anxiety disorders posttraumatic stress disorders that influences their underlying neurocircuitry. J. Affect. Disord..

[91] Lahey B.B. (2009). Public health significance of neuroticism. Am. Psychol..

[92] Diener E., Chan M.C. (2011). Happy people live longer: Subjective well-being contributes to health longevity. Appl Psychol. Health Well Being.

[93] Fredrickson B.L., Grewen K.M., Coffey K.A., Algoe S.B., Firestine A.M., Arevalo J.M., Ma J., Cole S.W. (2013). A functional genomic perspective on human well-being. Proc. Natl. Acad. Sci. USA.

[94] Walker E.A., Katon W., Russo J., Ciechanowski P., Newman E., Wagner A.W. (2003). Health care costs associated with posttraumatic stress disorder symptoms in women. Arch. Gen. Psychiatry.

[95] Kearns M.C., Ressler K.J., Zatzick D., Rothbaum B.O. (2012). Early interventions for PTSD: A review. Depres. Anxiety.

