# Supplementary material for: Predicting Health-Related Quality of Life in Trauma-Exposed Male Veterans in Late Midlife: A 20 Year Longitudinal Study
Source: Int J Environ Res Public Health. 2020 Jun 24;17(12):4554. doi: 10.3390/ijerph17124554 (PMC7345107; doi:10.3390/ijerph17124554)
Supplement: Supplementary file 1 [file ijerph-17-04554-s001.pdf]

International Journal of
Environmental Research and Public Health

MDPI

Supplemental Table S1. Partial Correlations between Neuroticism Polygenic Risk Scores at Different Thresholds, Posttraumatic Stress Symptoms and Covariates.

**Measure**	***p*-value threshold**			
	**0.01**	**0.05**	**0.1**	**0.5**
PTSS (SOH)	0.10013	**0.10315**	0.11442	0.10079
	0.009	**0.0071**	0.0028	0.0085
Age (SOH)	0.00282	**−0.00884**	0.00681	−0.01604
	0.9437	**0.8247**	0.8645	0.6875
Education	−0.00921	**−0.04681**	−0.03505	−0.02509
	0.8083	**0.2175**	0.3558	0.5087
Combat Exposure (SOH)	0.02898	**0.01073**	0.04167	0.00986
	0.4505	**0.7799**	0.2779	0.7974
Childhood Trauma	0.06259	**0.05019**	0.05448	0.06825
	0.0989	**0.186**	0.1511	0.072
Self-Rated Health (SOH)	0.00562	**0.03842**	0.03301	0.02996
	0.888	**0.3353**	0.4078	0.4525
Income (SOH)	0.00461	**0.00365**	0.03881	0.04239
	0.9081	**0.927**	0.3304	0.2876
Alcohol Consumption (SOH)	0.04878	**0.0512**	0.05004	0.0323
	0.2211	**0.199**	0.2094	0.4179
Health Problems (SOH)	0.05111	**0.03852**	0.03524	0.04363
	0.1998	**0.334**	0.3768	0.2739

Partial correlations adjust for the first 3 Principal Components representing ancestry; The bold column represents the $p = 0.05$ Polygenic risk score used in data analyses.; SOH—Survey of Health; PTSS—Posttraumatic Stress Symptoms.

Supplemental Table S2. Associations between Posttraumatic Stress Symptoms at Age 38 and Short Form 36 Mental HRQOL Subscales.

Measure	SF36 Vitality			SF36 Social Functioning			SF36 Role Emotional			SF36 Mental Health		
	Parameter Estimate (SE)	95% CI	*p*	Parameter estimate (SE)	95% CI	*p*	Parameter estimate (SE)	95% CI	*p*	Parameter estimate (SE)	95% CI	*p*
Intercept	11.82 (2.90)	[6.14, 17.50]	<0.0001	8.81 (1.29)	[6.28, 11.34]	<0.0001	5.07 (0.58)	[3.93, 6.21]	<0.0001	19.40 (3.03)	[13.47, 25.34]	<0.0001
PTSS	−0.13 (0.04)	[−0.21, −0.06]	0.0004	−0.07 (0.02)	[−0.11, −0.03]	0.0005	−0.02 (0.01)	[−0.04, −0.01]	0.0077	−0.16 (0.04)	[−0.25, −0.08]	0.0002
Time	0.16 (0.51)	[−0.84, 1.16]	0.7530	0.09 (0.20)	[−0.30, 0.48]	0.6629	0.14 (0.15)	[−0.15, 0.43]	0.3548	0.73 (0.55)	[−0.35, 1.80]	0.1876
PTSS * Time	−0.01 (0.02)	[−0.05, 0.04]	0.8099	−0.01 (0.01)	[−0.02, 0.01]	0.3544	−0.01 (0.01)	[−0.02, 0.01]	0.2280	−0.01 (0.02)	[−0.05, 0.04]	0.7595
Age	0.18 (0.06)	[0.05, 0.30]	0.0061	0.02 (0.03)	[−0.03, 0.07]	0.4563	0.02 (0.01)	[−0.01 ,0.04]	0.1868	0.22 (0.076)	[0.09, 0.35]	0.0008
Education	0.14 (0.07)	[0.01, 0.27]	0.0416	0.07 (0.03)	[0.01, 0.13]	0.0184	0.03 (0.01)	[−0.00 ,0.05]	0.0542	0.07 (0.07)	−0.08, 0.21]	0.3703
Combat Exposure	0.06 (0.05)	[−0.04, 0.16]	0.2189	−0.02 (0.02)	[−0.07, 0.02]	0.3294	−0.02 (0.01)	[−0.04, 0.01]	0.1736	−0.02 (0.06)	[−0.13, 0.10]	0.7880
Childhood Trauma	−0.16 (0.11)	[−0.37, 0.04]	0.1223	−0.10 (0.04)	[−0.18, −0.01]	0.0232	−0.06 (0.02)	[−0.10, −0.01]	0.0093	−0.27 (0.11)	[−0.48, −0.05]	0.0155
Self-rated Health	−0.76 (0.19)	[−1.12, −0.39]	<0.0001	−0.16 (0.08)	[−0.32, −0.01]	0.0399	−0.08 (0.04)	[−0.15, −0.01]	0.0299	−0.48 (0.19)	[−0.85, −0.11]	0.0109
Married Once	−0.45 (0.66)	[−1.74, 0.83]	0.4896	0.21 (0.25)	[−0.29, 0.71]	0.4093	0.23 (0.14)	[−0.04, 0.49]	0.0915	0.27 (0.63)	[−0.98, 1.51]	0.6746
Married > 1 Time	−0.36 (0.70)	[−1.72, 1.01]	0.6065	0.33 (0.27)	[−0.20, 0.86]	0.2228	0.29 (0.14)	[0.01, 0.56]	0.0437	0.61 (0.69)	[−0.74, 1.96]	0.3757
Income	0.22 (0.07)	[0.08, 0.35]	0.0015	0.08 (0.03)	[0.02, 0.14]	0.0103	0.03 (0.01)	[0.01, 0.06]	0.0204	0.15 (0.07)	[0.01, 0.29]	0.0301
Current Smoker	−0.87 (0.38)	[−1.61, −0.13]	0.0218	−0.04 (0.15)	[−0.34, 0.26]	0.7725	−0.05 (0.07)	[−0.20 ,0.09]	0.4593	−0.21 (0.36)	[−0.93, 0.50]	0.5594
Former Smoker	−0.39 (0.32)	[−1.01, 0.23]	0.2217	−0.22 (0.13)	[−0.48, 0.04]	0.0996	−0.12 (0.07)	[−0.25, 0.01]	0.0658	−0.48 (0.33)	[−1.13, 0.17]	0.1487
Alcohol Consumption	0.00 (0.01)	[−0.02, 0.03]	0.7166	−0.01 (0.01)	[−0.02, 0.01]	0.3381	0.00 (0.00)	[−0.00, 0.01]	0.5474	−0.00 (0.01)	[−0.02, 0.02]	0.9376
Health Problems	0.02 (0.23)	[−0.42, 0.47]	0.9170	−0.27 (0.12)	[−0.50, −0.03]	0.0254	−0.04 (0.06)	[−0.16, 0.07]	0.4586	−0.23 (0.25)	[−0.72, 0.27]	0.3646
Neuroticism PRS	−0.64 (0.44)	[−1.52, 0.23]	0.1477	−0.37 (0.22)	[−0.79, 0.05]	0.0874	−0.06 (0.09)	[−0.24, 0.12]	0.5062	−0.56 (0.48)	[−1.49, 0.37]	0.2394
PTSS*Neuroticism PRS	0.01 (0.02)	[−0.02, 0.05]	0.4677	0.01 (0.01)	[−0.01, 0.03]	0.2412	0.00 (0.00)	[−0.01, 0.01]	0.7492	0.00 (0.02)	[−0.04, 0.04]	0.9374
PC1	3.18 (3.77)	[−4.20, 10.56]	0.3983	1.96 (1.45)	[−0.88, 4.80]	0.1768	0.48 (0.73)	[−0.95, 1.91]	0.5115	3.61 (3.42)	[−3.10, 10.32]	0.2916
PC2	−3.51 (4.32)	[−11.97, 4.96]	0.4168	0.39 (1.70)	[−2.94, 3.73]	0.8170	−0.82 (0.87)	[−2.53, 0.90]	0.3500	−0.28 (4.31)	[−8.72, 8.16]	0.9487
PC3	−7.59 (3.38)	[−14.22, −0.97]	0.0247	−3.90 (1.30)	[−6.46, −1.35]	0.0028	−0.99 (0.72)	[−2.40, 0.43]	0.1713	−2.72 (3.45)	[−9.48, 4.04]	0.4300

Abbreviations: CI = Confidence Interval; PTSS = Posttraumatic Stress Symptoms; PRS = Polygenic Risk Score. PC (1–3) = first 3 principal components representing ancestry. PTSS, age, combat exposure, self-rated health, marriage, income, smoking, alcohol consumption and health problems were assessed at Survey of Health (average age 38). Time is a fixed effect and represents the time between VETSA 1 and VETSA 2 HRQOL assessments. Values at $p < 0.0063$ are significant for the Bonferroni corrected value for the 8 SF36 subscales at $p = 0.05/8 = 0.0063$ two-tailed. P-values shown are not Bonferroni corrected.

Supplemental Table S3. Associations between Posttraumatic Stress Symptoms at Age 38 and Short Form 36 Physical HRQOL Subscales.

	SF 36 General Health			SF 36 Bodily Pain			SF36 Physical Functioning			SF36 Role Physical		
**Measure**	**Parameter Estimate (SE)**	**95% CI**	***p***	**Parameter estimate (SE)**	**95% CI**	***p***	**Parameter estimate (SE)**	**95% CI**	***p***	**Parameter estimate (SE)**	**95% CI**	***p***
Intercept	16.07 (3.10)	[9.99, 22.15]	<0.0001	6.59 (1.47)	[3.70, 9.48]	<0.0001	25.74 (3.13)	[19.60, 31.87]	<0.0001	6.49 (0.98)	[4.57, 8.40]	<0.0001
PTSS	−0.07 (0.05)	[−0.17, 0.02]	0.1046	−0.05 (0.02)	[−0.08, −0.01]	0.0216	−0.07 (0.04)	[−0.15, 0.00]	0.0636	−0.03 (0.01)	[−0.06, −0.01]	0.008
Time	−0.06 (0.40)	[−0.86, 0.73]	0.8744	0.03 (0.28)	[−0.52, 0.58]	0.909	−1.27 (0.48)	[−2.22, −0.33]	0.0084	0.03 (0.21)	[−0.39, 0.45]	0.8836
PTSS * Time	−0.02 (0.02)	[−0.05, 0.02]	0.2828	−0.01 (0.01)	[−0.04, 0.01]	0.266	0.01 (0.02)	[−0.03, 0.05]	0.7671	−0.01 (0.01)	[−0.03, 0.01]	0.2817
Age	0.13 (0.07)	[−0.00, 0.25]	0.0553	0.07 (0.03)	[0.01, 0.13]	0.0321	0.04 (0.07)	[−0.09, 0.17]	0.5612	0.02 (0.02)	[−0.02, 0.07]	0.3056
Education	0.19 (0.08)	[0.04, 0.34]	0.0158	0.14 (0.04)	[0.06, 0.21]	0.0003	0.23 (0.07)	[0.09, 0.38]	0.0013	0.06 (0.02)	[0.01, 0.10]	0.01
Combat Exposure	−0.01 (0.05)	[−0.11, 0.10]	0.8975	−0.02 (0.03)	[−0.07, 0.03]	0.4947	0.00 (0.06)	[−0.11, 0.11]	0.9477	−0.02 (0.02)	[−0.06, 0.01]	0.197
Childhood Trauma	−0.29 (0.12)	[−0.52, −0.05]	0.0158	−0.14 (0.06)	[−0.25, −0.02]	0.0168	−0.16 (0.12)	[−0.39, 0.07]	0.1755	−0.07 (0.04)	[−0.15, −0.00]	0.0483
Self-rated Health	−1.42 (0.21)	[−1.83, −1.01]	<0.0001	−0.45 (0.10)	[−0.64, −0.26]	<0.0001	−0.76 (0.21)	[−1.17, −0.35]	0.0003	−0.16 (0.06)	[−0.28, −0.03]	0.0156
Married Once	−0.16 (0.66)	[−1.45, 1.12]	0.8015	−0.30 (0.30)	[−0.88, 0.28]	0.3138	−0.93 (0.55)	[−2.01, 0.15]	0.0915	−0.03 (0.19)	[−0.40, 0.35]	0.8943
Married > 1 Time	−0.44 (0.70)	[−1.82, 0.94]	0.5304	−0.28 (0.33)	[−0.92, 0.36]	0.3949	−1.29 (0.60)	[−2.46, −0.11]	0.0322	−0.13 (0.22)	[−0.55, 0.29]	0.5435
Income	0.18 (0.08)	[0.04, 0.33]	0.0152	0.10 (0.04)	[0.03, 0.17]	0.0063	0.18 (0.08)	[0.03, 0.33]	0.0184	0.07 (0.03)	[0.02, 0.12]	0.0093
Current Smoker	−1.31 (0.41)	[−2.11, −0.51]	0.0014	0.06 (0.20)	[−0.34, 0.46]	0.7746	−1.10 (0.43)	[−1.95, −0.26]	0.0107	−0.01 (0.13)	[−0.28, 0.25]	0.922
Former Smoker	−0.82 (0.33)	[−1.47, −0.18]	0.0119	−0.11 (0.17)	[−0.45, 0.22]	0.5135	−0.57 (0.32)	[−1.20, 0.06]	0.0781	−0.11 (0.11)	[−0.33, 0.11]	0.3391
Alcohol Consumption	0.01 (0.01)	[−0.02, 0.03]	0.595	−0.01 (0.01)	[−0.02, 0.01]	0.4301	−0.02 (0.02)	[−0.05, 0.01]	0.1797	−0.00 (0.01)	[−0.01, 0.01]	0.5118
Health Problems	−0.10 (0.25)	[−0.59, 0.39]	0.6833	−0.38 (0.17)	[−0.72, −0.04]	0.0297	−0.79 (0.30)	[−1.37, −0.21]	0.0074	−0.18 (0.10)	[−0.37, 0.01]	0.0598
Neuroticism PRS	−0.27 (0.53)	[−1.30, 0.77]	0.6156	−0.19 (0.22)	[−0.63, 0.25]	0.3919	−0.38 (0.51)	[−1.38, 0.62]	0.4569	−0.26 (0.14)	[−0.54, 0.02]	0.0727
PTSS*Neuroticism PRS	0.01 (0.02)	[−0.04, 0.05]	0.7335	0.01 (0.01)	[−0.01, 0.03]	0.4951	0.02 (0.02)	[−0.02, 0.06]	0.2716	0.01 (0.01)	[−0.00, 0.02]	0.0689
PC1	6.93 (4.03)	[−0.97, 14.84]	0.0857	0.58 (2.01)	[−3.36, 4.53]	0.7719	8.48 (3.81)	[1.02, 15.94]	0.0259	1.19 (1.31)	[−1.38, 3.75]	0.3649
PC2	−4.40 (4.57)	[−13.36, 4.56]	0.3357	−2.80 (2.08)	[−6.88, 1.28]	0.1784	1.46 (4.35)	[−7.07, 9.99]	0.7377	−1.46 (1.37)	[−4.14, 1.23]	0.2868
PC3	−8.71 (4.08)	[−16.70, −0.72]	0.0327	−5.82 (1.81)	[−9.37, −2.27]	0.0013	−9.72 (3.81)	[−17.20, −2.25]	0.0107	−3.24 (1.13)	[−5.46, −1.02]	0.0042

Abbreviations: CI = Confidence Interval; PTSS = Posttraumatic Stress Symptoms; PRS = Polygenic Risk Score.; PC (1–3) = first 3 principal components representing ancestry.; PTSS, age, combat exposure, self-rated health, marriage, income, smoking, alcohol consumption and health problems were assessed at Survey of Health (average age 38).; Time is a fixed effect and represents the time between VETSA 1 and VETSA 2 HRQOL assessments. Values at $p < 0.0063$ are significant for the Bonferroni corrected value for the 8 SF36 subscales at $p = 0.05/8 = 0.0063$ two-tailed. P-values shown are not Bonferroni corrected
